# Primary Tibiotalocalcaneal Nailing vs Open Reduction and Internal Fixation for Fragility Ankle Fractures in Older Adults: A Markov Model

**DOI:** 10.1177/24730114261450956

**Published:** 2026-05-31

**Authors:** Andrew Bouras, Akin A. Adio, Rahul Kumar, Rohan Phadke, Samuel W. Rice, Kush Mody, Anthony Ndu

**Affiliations:** 1 Nova Southeastern University Kiran C. Patel College of Osteopathic Medicine, FL, USA; 2 Perelman School of Medicine at the University of Pennsylvania, Philadelphia, PA, USA; 3 University of Massachusetts Chan Medical School, Worcester, MA, USA; 4 School of Medicine, Baylor College of Medicine, Houston, TX, USA; 5 Department of Orthopedics and Physical Rehabilitation, University of Massachusetts Chan Medical School, Worcester, MA, USA; 6 Department of Orthopaedics, Rutgers New Jersey Medical School, Newark, NJ, USA; 7 Department of Orthopaedic Surgery, Perelman School of Medicine at the University of Pennsylvania, Philadelphia, PA, USA

**Keywords:** Markov, tibiotalocalcaneal nailing, ankle ORIF, fragility ankle fractures

## Abstract

**Background:**

Fragility ankle fractures are common in the elderly population. The cost-effectiveness of primary tibiotalocalcaneal (TTC) nailing compared with open reduction and internal fixation (ORIF) in this population remains unclear.

**Methods:**

We constructed a Markov cohort model with a 4-year time horizon to compare TTC nailing vs ORIF for fragility ankle fractures in patients aged 75 years and older. All complication rates were derived from the McDonald et al’s (2025) systematic review and meta-analysis. Costs were in 2024 US dollars with 3% annual discounting for costs and outcomes. Probabilistic and deterministic sensitivity analyses were performed.

**Results:**

In the base case, ORIF yielded 3.210 quality-adjusted life years (QALYs) (95% credible interval [CrI] 2.785-3.584) at $30 091 (95% CrI $21 426-$40 852) compared with 3.207 QALYs (95% CrI 2.783-3.581) at $33 583 (95% CrI $23 741-$45 447) for TTC nailing. ORIF dominated incremental cost (+$3492, 95% CrI −$11 467 to +$18 273) and incremental QALYs (−0.003, 95% CrI −0.009 to +0.002). The cost differential was driven by the $3000 higher index procedure cost for TTC, partially offset by lower superficial infection (2.1% vs 10.2%). Probabilistic sensitivity analysis demonstrated 33% probability of cost-effectiveness at $100 000/QALY. All 4 scenario analyses confirmed that ORIF dominated.

**Conclusion:**

Under McDonald’s pooled estimates, TTC nailing is dominated by ORIF for fragility ankle fractures in older adults. The lower superficial infection rate with TTC does not offset its higher procedure cost and higher rates of nonunion and hardware failure. These findings support ORIF as the preferred strategy from a cost-effectiveness standpoint, although the small incremental differences should be considered alongside clinical factors when selecting treatment.

**Level of Evidence:**

Level IV, economic modeling.

## Introduction

Fragility ankle fractures are low-energy injuries that occur in osteoporotic bone and present substantial clinical challenges.^1,2^ Open reduction and internal fixation (ORIF) have traditionally been the standard treatment for unstable ankle fractures. However, in older adults, ORIF is associated with high rates of wound complications and deep infection, which may exceed 20% in high-risk cohorts.^3^ Primary tibiotalocalcaneal (TTC) nailing has emerged as an alternative treatment for fragile elderly patients with ankle fractures.^4^ This technique provides stable fixation while permitting early full weight bearing and preserving the soft-tissue envelope in patients with limited ambulatory demand and multiple comorbidities. The percutaneous nature of TTC nailing reduces the risk of surgical site infection and mitigates functional decline associated with prolonged non-weight-bearing.^5-7^

However, the higher upfront cost of intramedullary implants, the economic impact of reduced complication rates, and improved discharge disposition remains poorly defined. No previous study has performed a rigorous cost-utility analysis comparing these 2 modalities in a geriatric cohort using contemporary evidence. We therefore conducted a cost-effectiveness analysis using a Markov state-transition model to compare primary TTC nailing and ORIF for fragility ankle fractures in patients aged 75 years and older.

## Methods

### Introduction to Markov Modeling

Markov state-transition models are commonly used in health economic analyses to evaluate clinical and economic outcomes by simulating transitions between discrete health states over time.^8^ Each health state is assigned an associated cost and quality-of-life value. Patients transition between states according to predefined probabilities that reflect the likelihood of clinical events occurring during a fixed cycle length. These transition probabilities are derived from published literature, registry data, or expert consensus when high-quality evidence is unavailable. Quality of life is measured on a scale from 0, representing death, to 1, representing perfect health, and is typically expressed using validated instruments such as the EuroQol–5 dimensions.^9^ During each cycle, patients accrue quality-adjusted life-years (QALYs) and costs based on the health state they occupy. Over the full model horizon, cumulative costs and QALYs are calculated for each treatment strategy. The primary outcome of a Markov cost-utility analysis is the incremental cost-effectiveness ratio, defined as the difference in cost between 2 strategies divided by the difference in QALYs gained. In this study, the strategies compared were TTC and ORIF for the treatment of fragility ankle fractures in older adults. The analysis was conducted from a health care payer perspective, incorporating direct medical costs derived from CMS reimbursement data. A willingness-to-pay threshold represents the maximum amount society is willing to pay for 1 additional QALY, typically set at US$100 000. Strategies with an incremental cost-effectiveness ratio below this threshold are considered cost-effective, whereas strategies that result in lower costs and greater QALYs are considered dominant.

### Model Structure

The Markov decision tree model used in this study was constructed from publicly available software (TreeAge Pro 2025, R2; TreeAge Software).^10^ Each patient in the model was assumed to be an older adult with a fragility ankle fracture undergoing operative treatment, without preexisting ipsilateral amputation or active infection at the time of surgery. All patients were assumed to undergo definitive fixation with 1 of 2 treatment strategies: TTC nailing or ORIF. Patients entered the model at the time of hospital discharge following index surgical treatment.

**Figure 1.**
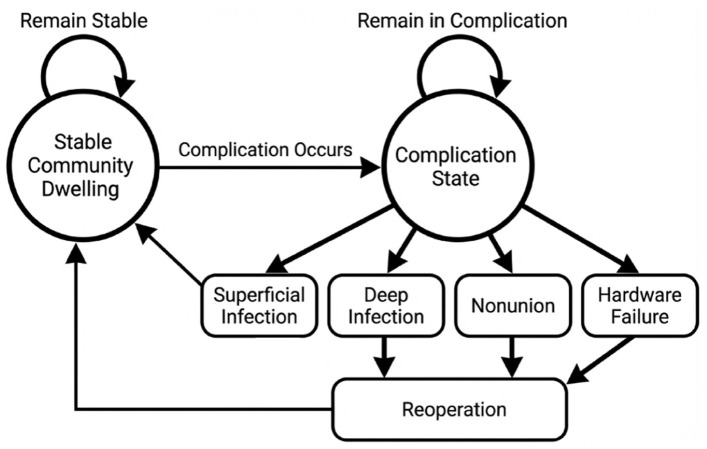
Markov state-transition diagram.

In Markov modeling, time is divided into discrete intervals known as cycles, during which patients transition between health states based on derived probabilities. Prior clinical studies indicate that the majority of complications following surgical treatment of fragility ankle fractures occur early in the postoperative course.^11^ For this reason, quarterly cycle lengths were used during the first postoperative year to capture acute complications and their impact on quality of life, followed by annual cycles thereafter. Specifically, the model used 4 quarterly cycles during the first postoperative year, followed by annual cycles for years 2 through 5. The model consisted of 3 mutually exclusive health states: stable community dwelling, superficial infection, and reoperation. Following index treatment, patients could either remain in a stable state or develop a complication. Patients experiencing superficial infection entered a temporary health state and returned to stable community dwelling after treatment. Patients with deep infection, nonunion, or hardware failure entered a reoperation pathway, reflecting the shared need for surgical intervention and similar downstream costs and quality-of-life impacts. Within each model cycle, patients were limited to a single complication pathway, after which they transitioned to the corresponding post-treatment state. A 4-year time horizon was selected to reflect the mean follow-up of the McDonald et al^6^ meta-analysis (approximately 46 months), and to capture the majority of complications, reoperations, and associated costs, which typically occur within the early postoperative period in this elderly population with limited life expectancy. Costs and health outcomes were discounted at an annual rate of 3% in accordance with recommendations of the Second Panel on Cost-Effectiveness in Health and Medicine.^12^ An overview of the model structure is shown in Figure 1.

### Data Sources

Clinical efficacy parameters were derived from the pooled estimates reported in the meta-analysis by McDonald et al.^6^ Health state utilities were obtained from van Gerven et al,^14^ and cost inputs were derived from Centers for Medicare and Medicaid Services (CMS) reimbursement data.^13^ All model input parameters and sources are summarized in Table 1. In this model, non-absorbing states represent temporary conditions from which patients may recover, whereas absorbing states represent stable functional states without further transition.

**Table 1.** Comprehensive Model Input Parameters.

Parameter	Base Case	SA Range (±20%)	Distribution	Source
Clinical complication rates, %				
Superficial infection				
ORIF	10.20	8.2-12.2	Beta	McDonald 2025^6^
TTC	2.10	1.7-2.5	Beta	
Deep infection				
ORIF	2.50	2.0-3.0	Beta	
TTC	3.43	2.7-4.1	Beta	
Nonunion				
ORIF	11.70	8.4-15	Beta	
TTC	5.30	3.8-6.8	Beta	
Hardware failure				
ORIF	6.60	4.8-8.4	Beta	
TTC	8.80	6.8-10.8	Beta	
Costs, USD (2024)				
Index procedure, ORIF	25 000	20 000-30 000	Gamma	CMS DRG 509/511, 2024 IPPS^13^
Index procedure, TTC	28 000	22 400-33 600	Gamma	CMS DRG 509/511^13^ + implant premium^9^
Superficial infection treatment	2500	2000-3000	Gamma	CPT 10060/10061, CMS PFS 2024^13^
Utilities, EQ-5D score				
Age-adjusted baseline (age 75 y)	0.9	0.8-1.0	Fixed	van Gerven et al.^14^
Temporary reoperation quarter cycle utility	0.78	0.68-0.88	Beta	van Gerven et al.^14^

Abbreviations: CMS, Centers for Medicare & Medicaid Services; *CPT*, *Current Procedural Terminology*; DRG, Diagnosis-Related Group; EQ-5D, EuroQol–5 dimensions; ORIF, open reduction and internal fixation; PFS, Physician Fee Schedule; SA, Sensitivity Analysis; TTC, tibiotalocalcaneal.

### Statistical Analysis

Transition probabilities and utilities were sampled from beta distributions, costs from gamma distributions, and hazard ratios from log-normal distributions. A total of 10 000 Monte Carlo iterations was performed. One-way tornado sensitivity analysis varied all key parameters by ±20%. One-way sensitivity analyses were performed using ±20% variation for parameters lacking reported CIs. This range was selected to approximate moderate parameter uncertainty commonly observed in clinical and cost estimates, while avoiding overly broad assumptions that could distort model stability. Probabilistic sensitivity analysis (PSA) used beta distributions (probabilities and utilities) and gamma distributions (costs). Cost-effectiveness acceptability curves were generated across willingness-to-pay thresholds from $0 to $300 000/QALY.

## Results

### Base-Case Findings

**Figure 2.**
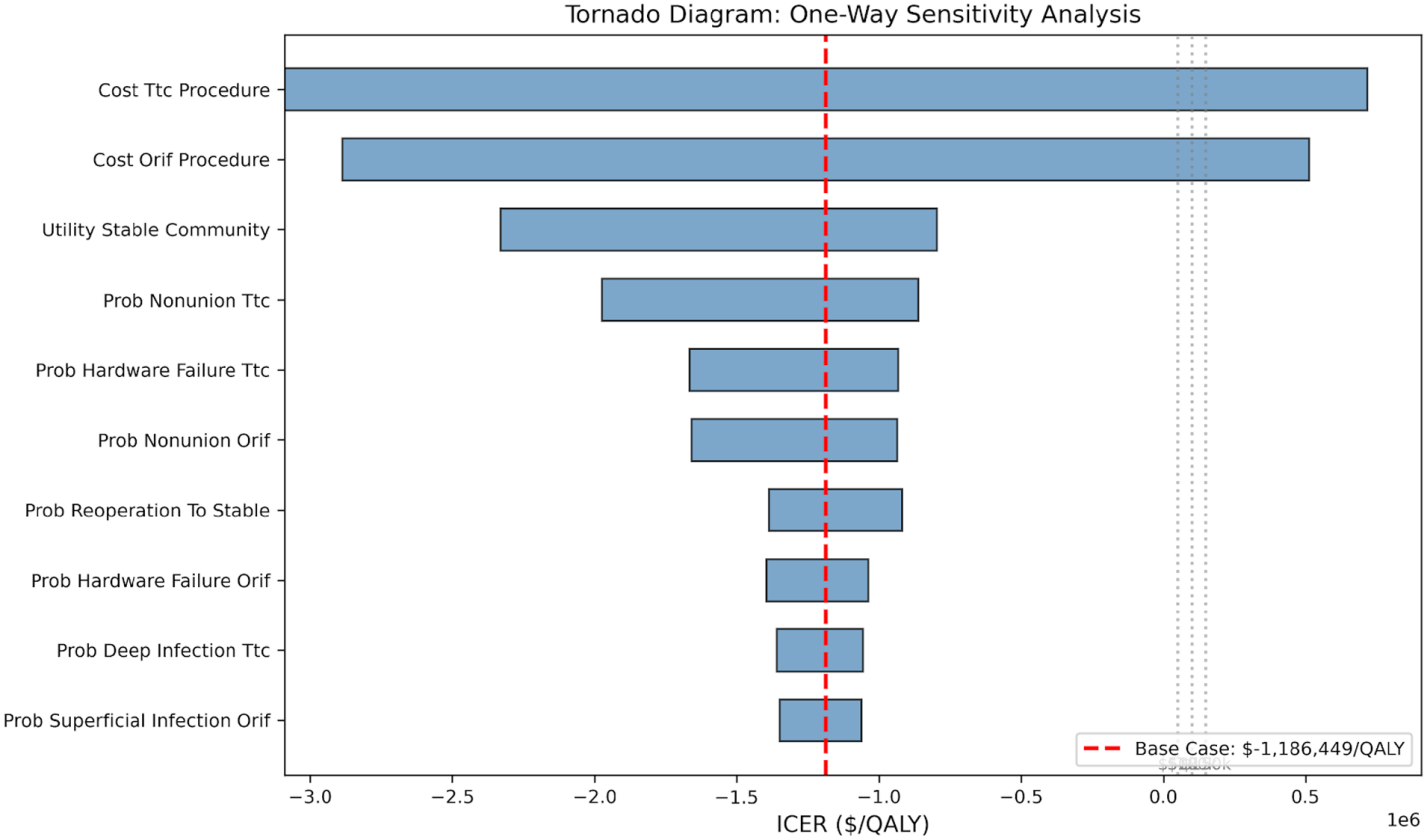
Tornado diagram. One-way sensitivity analysis showing the model is most sensitive to index procedure costs, stable community utility, and TTC nonunion and hardware failure rates.

In the base case, TTC nailing was dominated by ORIF—it was both more costly and less effective (Table 2). ORIF yielded 3.210 discounted QALYs (95% credible interval [CrI] 2.785-3.584) at a total discounted cost of $30 091 (95% CrI $21 426-$40 852), compared with 3.207 QALYs (95% CrI 2.783-3.581) at $33 583 (95% CrI $23 741-$45 447) for TTC nailing. The incremental cost of TTC was $3492 (95% CrI −$11 467 to +$18 273) with a QALY loss of 0.003 (95% CrI −0.009 to +0.002). All CrIs are derived from the 2.5th-97.5th percentiles of 10 000 PSA iterations. The cost differential was driven primarily by the $3000 higher index procedure cost for TTC nailing, with small additional cost differences arising from complication management (Figure 2).

**Table 2.** Base-Case Cost-Effectiveness Results.

Strategy	Total Discounted Costs, $ (95% CrI)	Total Discounted QALYs (95% CrI)	Incremental Cost, $ (95% CrI)	Incremental QALY (95% CrI)	CE Quadrant
ORIF	30 091 (21 426 to 40 852)	3.210 (2.785 to 3.584)	Ref	Ref	Ref
TTC Nail	33 583 (23 741 to 45 447)	3.207 (2.783 to 3.581)	+3492 (−11 467 to +18 273)	−0.003 (−0.009 to +0.002)	Dominated

Abbreviations: CE, Cost Effectiveness; CrI, credible interval; ORIF, open reduction and internal fixation; QALY, quality-adjusted life year; TTC, tibiotalocalcaneal.

### Sensitivity Analysis

**Figure 3.**
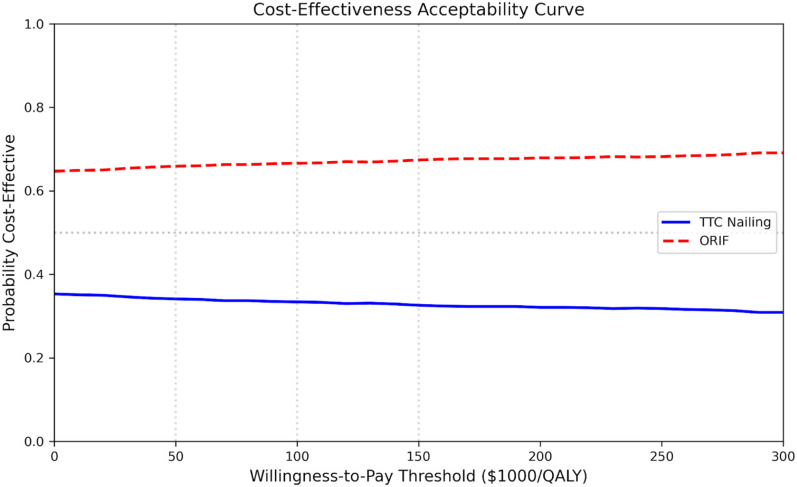
Cost-effectiveness acceptability curve. The probability of TTC cost-effectiveness is approximately 33% across $50 000-$150 000/QALY thresholds, reflecting substantial parameter uncertainty.

**Figure 4.**
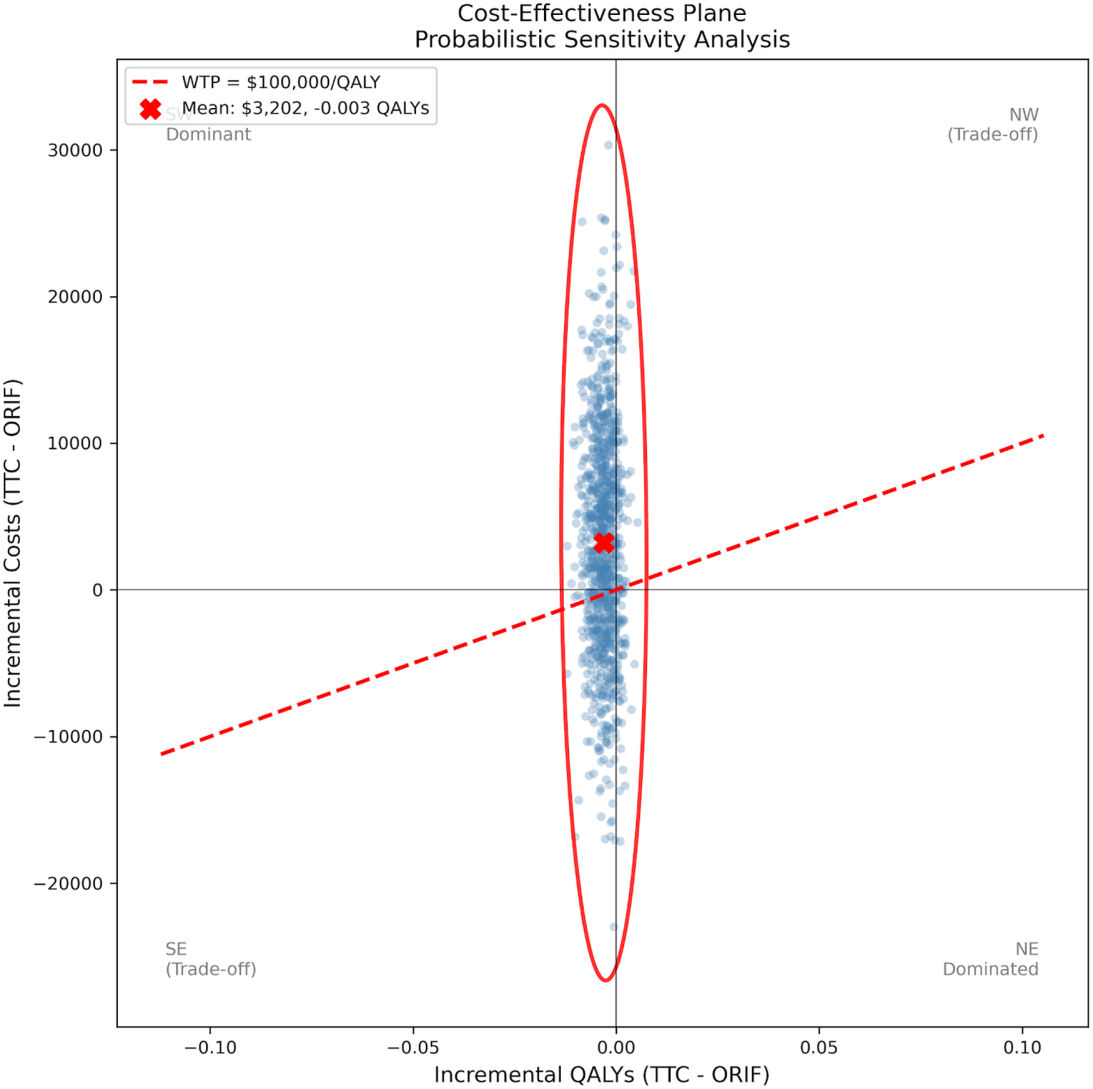
Cost-effectiveness plane. Distribution of 1000 PSA iterations showing incremental costs and QALYs of TTC vs ORIF. The red dashed line represents the $100 000/QALY willingness-to-pay threshold. Approximately 33% of iterations fall below the WTP threshold.

The most influential parameters were the index procedure costs (TTC and ORIF), stable community utility, TTC nonunion rate, and TTC hardware failure rate (Figure 2). Notably, no single parameter variation reversed the dominated finding within the ±20% range tested. Of 10,000 PSA iterations, TTC was cost-effective in approximately 33% of iterations at a willingness-to-pay threshold of $100 000/QALY (Figure 3). This probability was stable across WTP thresholds, ranging from 34% at $50 000/QALY to 33% at $150 000/QALY. The wide uncertainty reflects the small base-case incremental differences and substantial parameter uncertainty (Figure 4).

### Scenario Analyses

Scenario analyses were performed to test the robustness of the base-case findings under alternative clinical and modeling assumptions (Table 3). Across all scenarios TTC nailing remained dominated by ORIF, with higher costs and lower quality-adjusted life years (QALYs). In the equal deep infection scenario, where deep infection rates were set to 2.5% for both strategies, TTC remained dominated with an incremental cost of $3425 and a QALY loss of 0.002 compared with ORIF. Similarly, when a more conservative 3-year time horizon was applied, TTC continued to be dominated, with an incremental cost of $3486 and a QALY decrement of 0.003. Increasing all reoperation transition probabilities by 50% also did not alter the results, yielding an incremental cost of $3493 and a QALY loss of 0.002 for TTC relative to ORIF.

**Table 3.** Scenario Analyses.

Scenario	Incremental Cost, $	Incremental QALY	CE Quadrant
Base case	+3492	−0.003	Dominated
Equal deep infection (both 2.5%)	+3425	−0.002	Dominated
3-y time horizon	+3486	−0.003	Dominated
Higher reoperation (+50% in both arms)	+3493	−0.002	Dominated

Abbreviations: CE, Cost Effectiveness; QALY, quality-adjusted life year.

## Discussion

Few US-based cost-effectiveness analyses have evaluated surgical treatment strategies for geriatric ankle fractures, and to our knowledge, none have directly compared TTC nailing with ORIF in this population. This study suggests that ORIF represents a dominant economic strategy compared with TTC for geriatric fragility ankle fractures. ORIF was associated with both lower costs and modestly higher health utility across multiple sensitivity analyses. Within this context, the incremental QALY gain of 0.003 observed with ORIF is modest but typical for surgical comparisons in geriatric fragility fractures.^15,16^ However, the observed advantages of ORIF in this analysis were driven primarily by differences in cost rather than large differences in health utility. According to our analysis, treating 1000 patients with TTC rather than ORIF would be expected to result in approximately 3 fewer quality-adjusted life years and about $3.5 million in additional aggregate costs.

Much of the rationale for TTC nailing in geriatric ankle fractures relates to its minimally invasive technique and reduced soft tissue dissection. TTC constructs avoid extensive surgical exposure and may therefore reduce wound complications in medically complex patients with poor soft-tissue envelopes.^4^ McDonald et al^6^ reported a lower rate of superficial infection with TTC nailing compared with ORIF (2.1% vs 10.2%) in their meta-analysis of geriatric ankle fractures. However, other clinical outcomes appear broadly comparable between the 2 techniques. Georgiannos et al^7^ found similar functional scores and return to baseline mobility between TTC and ORIF despite differences in postoperative complications and hospital length of stay. Similarly, systematic reviews of TTC fixation have reported acceptable union and complication rates but no consistent superiority over traditional fixation methods. These findings suggest that although TTC may reduce certain soft-tissue complications, the overall clinical effectiveness of the procedures remains broadly similar.

One factor not incorporated into the model was the potential impact of earlier mobilization associated with TTC constructs. TTC fixation provides load-sharing stabilization across the ankle and hindfoot and may permit earlier weight bearing than traditional ORIF constructs.^17^ Georgiannos et al^7^ reported shorter hospital stays in patients treated with TTC compared with ORIF, highlighting a potential benefit related to earlier postoperative mobility. However, these outcomes remain difficult to quantify across the existing literature. Studies evaluating TTC fixation consist of small case series or heterogeneous patient populations, and outcomes such as length of stay, discharge disposition, and time to functional recovery are reported inconsistently.^6^ Because these parameters could not be reliably estimated, they were not incorporated into the model. Although earlier mobilization may confer downstream economic benefits, the variability in the available evidence limits robust estimation of these effects in cost-effectiveness analyses.

### Limitations

This study has several limitations. First, complication rates were derived from pooled estimates of a meta-analysis and may not reflect patient-level differences, surgeon experience, or institutional variation. This may limit the ability of the model to capture heterogeneity in outcomes across different clinical settings. Second, health state utilities were obtained from published fracture cohorts rather than studies directly comparing TTC and ORIF, requiring assumptions about functional differences between treatments. As a result, the modeled quality-of-life differences between procedures may not fully represent procedure-specific outcomes. Additionally, cost inputs were based on CMS reimbursement estimates and may not represent variation in institutional pricing or implant costs. This may affect the generalizability of the economic results across health care systems or hospitals with different cost structures. Finally, length of hospital stay and time to return to mobility were not included because these outcomes were reported inconsistently across studies. Excluding these factors may underestimate potential economic effects related to postoperative recovery and early mobilization.

## Conclusion

TTC nailing was not cost-effective compared with ORIF in the base-case analysis, as it was associated with higher costs and slightly lower QALYs. Although the difference in effectiveness was small, the increased cost of TTC drove the overall result. Sensitivity and scenario analyses demonstrated that this finding remained robust across a wide range of assumptions, with TTC becoming cost-effective in only a minority of simulations at a willingness-to-pay threshold of $100 000/QALY. These results suggest that, in most cases, ORIF represents the more economically favorable strategy for fragility ankle fractures in elderly patients.

## Supplemental Material

sj-pdf-1-fao-10.1177_24730114261450956 – Supplemental material for Primary Tibiotalocalcaneal Nailing vs Open Reduction and Internal Fixation for Fragility Ankle Fractures in Older Adults: A Markov Model Supplemental material, sj-pdf-1-fao-10.1177_24730114261450956 for Primary Tibiotalocalcaneal Nailing vs Open Reduction and Internal Fixation for Fragility Ankle Fractures in Older Adults: A Markov Model by Andrew Bouras, Akin A. Adio, Rahul Kumar, Rohan Phadke, Samuel W. Rice, Kush Mody and Anthony Ndu in Foot & Ankle Orthopaedics
