# Supplementary material for: Primary Tibiotalocalcaneal Nailing vs Open Reduction and Internal Fixation for Fragility Ankle Fractures in Older Adults: A Markov Model
Source: Foot Ankle Orthop. 2026 May 31;11(2):24730114261450956. doi: 10.1177/24730114261450956 (PMC13226974; doi:10.1177/24730114261450956)
Supplement: sj-pdf-1-fao-10.1177_24730114261450956 – Supplemental material for Primary Tibiotalocalcaneal Nailing vs Open Reduction and Internal Fixation for Fragility Ankle Fractures in Older Adults: A Markov Model [file sj-pdf-1-fao-10.1177_24730114261450956.pdf]

Conflict of Interest (COI) Disclosure Form FAI / FAO

Purpose:
All authors submitting a manuscript to this peer-reviewed journal **must disclose any conflicts of interest (COI) related to the submitted paper**.
No other disclosures are required or desired.

A conflict of interest typically involves **financial relationships (most commonly payments to surgeons or authors)** that could reasonably be perceived as influencing the submitted work, whether **received in the past or anticipated in the future**.

**Author Name:**	Andrew Bouras
**Manuscript Title:**	**Lifetime Cost-Utility of Primary Tibiotalocalcaneal Nailing versus Open Reduction and Internal Fixation for Fragility Ankle Fractures in Older Adults: A Markov Model from the US Payer Perspective**
**Date:**	**2/11/26**

Conflict of Interest Disclosure:

Do you or a family member have conflicts of interest related to this manuscript or its subject matter?
(Examples include consulting fees, honoraria, royalties, stock or stock options, research funding, paid advisory roles, product or material support, or intellectual property interests (e.g., potential future royalties) valued over $500, received within the past three years or expected in the future.)

☒ **No — I have no conflicts of interest to disclose.**

☐ **Yes — I have a conflict of interest to disclose.**
If **Yes**, please briefly describe the financial relationship(s) below, including the company/entity involved and the nature of the payment or support:

Click or tap here to enter text.

Updates: If a relevant conflict of interest arises or changes after submission and before publication, an updated disclosure must be provided.

Publication of Disclosure: All disclosed conflicts of interest will be published with the article and completed forms will be made available as an online supplement.

Conflict of Interest (COI) Disclosure Form FAI / FAO

Purpose:
All authors submitting a manuscript to this peer-reviewed journal **must disclose any conflicts of interest (COI) related to the submitted paper**.
No other disclosures are required or desired.

A conflict of interest typically involves **financial relationships (most commonly payments to surgeons or authors)** that could reasonably be perceived as influencing the submitted work, whether **received in the past or anticipated in the future**.

**Author Name:**	Akin Adio
**Manuscript Title:**	**Lifetime Cost-Utility of Primary Tibiotalocalcaneal Nailing versus Open Reduction and Internal Fixation for Fragility Ankle Fractures in Older Adults: A Markov Model from the US Payer Perspective**
**Date:**	**2/11/26**

Conflict of Interest Disclosure:

Do you or a family member have conflicts of interest related to this manuscript or its subject matter?
(Examples include consulting fees, honoraria, royalties, stock or stock options, research funding, paid advisory roles, product or material support, or intellectual property interests (e.g., potential future royalties) valued over $500, received within the past three years or expected in the future.)

☒ **No — I have no conflicts of interest to disclose.**

☐ **Yes — I have a conflict of interest to disclose.**
If **Yes**, please briefly describe the financial relationship(s) below, including the company/entity involved and the nature of the payment or support:

Click or tap here to enter text.

Updates: If a relevant conflict of interest arises or changes after submission and before publication, an updated disclosure must be provided.

Publication of Disclosure: All disclosed conflicts of interest will be published with the article and completed forms will be made available as an online supplement.

Conflict of Interest (COI) Disclosure Form FAI / FAO

Purpose:
All authors submitting a manuscript to this peer-reviewed journal **must disclose any conflicts of interest (COI) related to the submitted paper**.
No other disclosures are required or desired.

A conflict of interest typically involves **financial relationships (most commonly payments to surgeons or authors)** that could reasonably be perceived as influencing the submitted work, whether **received in the past or anticipated in the future**.

**Author Name:**	Rahul Kumar
**Manuscript Title:**	**Lifetime Cost-Utility of Primary Tibiotalocalcaneal Nailing versus Open Reduction and Internal Fixation for Fragility Ankle Fractures in Older Adults: A Markov Model from the US Payer Perspective**
**Date:**	**2/11/26**

Conflict of Interest Disclosure:

Do you or a family member have conflicts of interest related to this manuscript or its subject matter?
(Examples include consulting fees, honoraria, royalties, stock or stock options, research funding, paid advisory roles, product or material support, or intellectual property interests (e.g., potential future royalties) valued over $500, received within the past three years or expected in the future.)

☒ **No — I have no conflicts of interest to disclose.**

☐ **Yes — I have a conflict of interest to disclose.**
If **Yes**, please briefly describe the financial relationship(s) below, including the company/entity involved and the nature of the payment or support:

Click or tap here to enter text.

Updates: If a relevant conflict of interest arises or changes after submission and before publication, an updated disclosure must be provided.

Publication of Disclosure: All disclosed conflicts of interest will be published with the article and completed forms will be made available as an online supplement.

Conflict of Interest (COI) Disclosure Form FAI / FAO

Purpose:
All authors submitting a manuscript to this peer-reviewed journal **must disclose any conflicts of interest (COI) related to the submitted paper**.
No other disclosures are required or desired.

A conflict of interest typically involves **financial relationships (most commonly payments to surgeons or authors)** that could reasonably be perceived as influencing the submitted work, whether **received in the past or anticipated in the future**.

**Author Name:**	Rohan Phadke
**Manuscript Title:**	**Lifetime Cost-Utility of Primary Tibiotalocalcaneal Nailing versus Open Reduction and Internal Fixation for Fragility Ankle Fractures in Older Adults: A Markov Model from the US Payer Perspective**
**Date:**	**2/11/26**

Conflict of Interest Disclosure:

Do you or a family member have conflicts of interest related to this manuscript or its subject matter?
(Examples include consulting fees, honoraria, royalties, stock or stock options, research funding, paid advisory roles, product or material support, or intellectual property interests (e.g., potential future royalties) valued over $500, received within the past three years or expected in the future.)

☒ **No — I have no conflicts of interest to disclose.**

☐ **Yes — I have a conflict of interest to disclose.**
If **Yes**, please briefly describe the financial relationship(s) below, including the company/entity involved and the nature of the payment or support:

Click or tap here to enter text.

Updates: If a relevant conflict of interest arises or changes after submission and before publication, an updated disclosure must be provided.

Publication of Disclosure: All disclosed conflicts of interest will be published with the article and completed forms will be made available as an online supplement.

Conflict of Interest (COI) Disclosure Form FAI / FAO

Purpose:
All authors submitting a manuscript to this peer-reviewed journal **must disclose any conflicts of interest (COI) related to the submitted paper**.
No other disclosures are required or desired.

A conflict of interest typically involves **financial relationships (most commonly payments to surgeons or authors)** that could reasonably be perceived as influencing the submitted work, whether **received in the past or anticipated in the future**.

**Author Name:**	Samuel W. Rice
**Manuscript Title:**	**Lifetime Cost-Utility of Primary Tibiotalocalcaneal Nailing versus Open Reduction and Internal Fixation for Fragility Ankle Fractures in Older Adults: A Markov Model from the US Payer Perspective**
**Date:**	**2/11/26**

Conflict of Interest Disclosure:

Do you or a family member have conflicts of interest related to this manuscript or its subject matter?
(Examples include consulting fees, honoraria, royalties, stock or stock options, research funding, paid advisory roles, product or material support, or intellectual property interests (e.g., potential future royalties) valued over $500, received within the past three years or expected in the future.)

☒ **No — I have no conflicts of interest to disclose.**

☐ **Yes — I have a conflict of interest to disclose.**
If **Yes**, please briefly describe the financial relationship(s) below, including the company/entity involved and the nature of the payment or support:

Click or tap here to enter text.

Updates: If a relevant conflict of interest arises or changes after submission and before publication, an updated disclosure must be provided.

Publication of Disclosure: All disclosed conflicts of interest will be published with the article and completed forms will be made available as an online supplement.

Conflict of Interest (COI) Disclosure Form FAI / FAO

Purpose:
All authors submitting a manuscript to this peer-reviewed journal **must disclose any conflicts of interest (COI) related to the submitted paper**.
No other disclosures are required or desired.

A conflict of interest typically involves **financial relationships (most commonly payments to surgeons or authors)** that could reasonably be perceived as influencing the submitted work, whether **received in the past or anticipated in the future**.

**Author Name:**	Kush Mody
**Manuscript Title:**	**Lifetime Cost-Utility of Primary Tibiotalocalcaneal Nailing versus Open Reduction and Internal Fixation for Fragility Ankle Fractures in Older Adults: A Markov Model from the US Payer Perspective**
**Date:**	**2/11/26**

Conflict of Interest Disclosure:

Do you or a family member have conflicts of interest related to this manuscript or its subject matter?
(Examples include consulting fees, honoraria, royalties, stock or stock options, research funding, paid advisory roles, product or material support, or intellectual property interests (e.g., potential future royalties) valued over $500, received within the past three years or expected in the future.)

☒ **No — I have no conflicts of interest to disclose.**

☐ **Yes — I have a conflict of interest to disclose.**
If **Yes**, please briefly describe the financial relationship(s) below, including the company/entity involved and the nature of the payment or support:

Click or tap here to enter text.

Updates: If a relevant conflict of interest arises or changes after submission and before publication, an updated disclosure must be provided.

Publication of Disclosure: All disclosed conflicts of interest will be published with the article and completed forms will be made available as an online supplement.

Conflict of Interest (COI) Disclosure Form FAI / FAO

Purpose:
All authors submitting a manuscript to this peer-reviewed journal **must disclose any conflicts of interest (COI) related to the submitted paper**.
No other disclosures are required or desired.

A conflict of interest typically involves **financial relationships (most commonly payments to surgeons or authors)** that could reasonably be perceived as influencing the submitted work, whether **received in the past or anticipated in the future**.

**Author Name:**	**Anthony Ndu**
**Manuscript Title:**	**Lifetime Cost-Utility of Primary Tibiotalocalcaneal Nailing versus Open Reduction and Internal Fixation for Fragility Ankle Fractures in Older Adults: A Markov Model from the US Payer Perspective**
**Date:**	**2/11/26**

Conflict of Interest Disclosure:

Do you or a family member have conflicts of interest related to this manuscript or its subject matter?
(Examples include consulting fees, honoraria, royalties, stock or stock options, research funding, paid advisory roles, product or material support, or intellectual property interests (e.g., potential future royalties) valued over $500, received within the past three years or expected in the future.)

☐ **No — I have no conflicts of interest to disclose.**

☒ **Yes — I have a conflict of interest to disclose.**
If **Yes**, please briefly describe the financial relationship(s) below, including the company/entity involved and the nature of the payment or support:

Enovis - Surgeon Advisory Board; Smith and Nephew - Surgeons Speaker Bureau; Kuros Biologics - Consultant

Updates: If a relevant conflict of interest arises or changes after submission and before publication, an updated disclosure must be provided.

Publication of Disclosure: All disclosed conflicts of interest will be published with the article and completed forms will be made available as an online supplement.
